# Correction: The myeloid cell-driven transdifferentiation of endothelial cells into pericytes promotes the restoration of BBB function and brain self-repair after stroke

**DOI:** 10.7554/eLife.112376

**Published:** 2026-06-17

**Authors:** Tingbo Li, Ling Yang, Jiaqi Tu, Yufan Hao, Zhu Zhu, Yingjie Xiong, Qingzhu Gao, Lili Zhou, Guanglei Xie, Dongdong Zhang, Xuzhao Li, Yuxiao Jin, Yiyi Zhang, Bingrui Zhao, Nan Li, Xi Wang, Jie-Min Jia

**Keywords:** Mouse

Li T, Yang L, Tu J, Hao Y, Zhu Z, Xiong Y, Gao Q, Zhou L, Xie G, Zhang D, Li X, Jin Y, Zhang Y, Zhao B, Li N, Wang X, Jia J-M. 2025. The myeloid cell-driven transdifferentiation of endothelial cells into pericytes promotes the restoration of BBB function and brain self-repair after stroke. *eLife* **14**:RP105593. doi: 10.7554/eLife.105593.

Published 16 July 2025

We have identified errors in the chart legend in Figure 9D of our published eLife article. One of the groups is incorrectly labelled. The order of the groups in the chart legend is incorrect and inconsistent with the other charts in Figure 9.

**How the correction was made**:

1. Group designation error (incorrect group names):

The experimental group (red) was incorrectly labelled “iECs”. The label has now been corrected to “iECs:Tgfbr2fl/fl”.

2. Incorrect placement of group positions:

The control group “Tgfbr2fl/fl” was incorrectly placed at the bottom of the chart legend, we have corrected this to place this at the top. In turn the experimental group “iECs:Tgfbr2fl/fl” was incorrectly placed at the top, we have corrected this to place this group at the bottom.

We have carefully rechecked the published eLife article. Importantly, these corrections do not affect the data presented in Figure 9, nor do they alter the results or conclusions of the study. All authors have reviewed and agreed to the changes.

The corrected Figure 9 (updated for panel D) is shown here:

**Figure 1:**
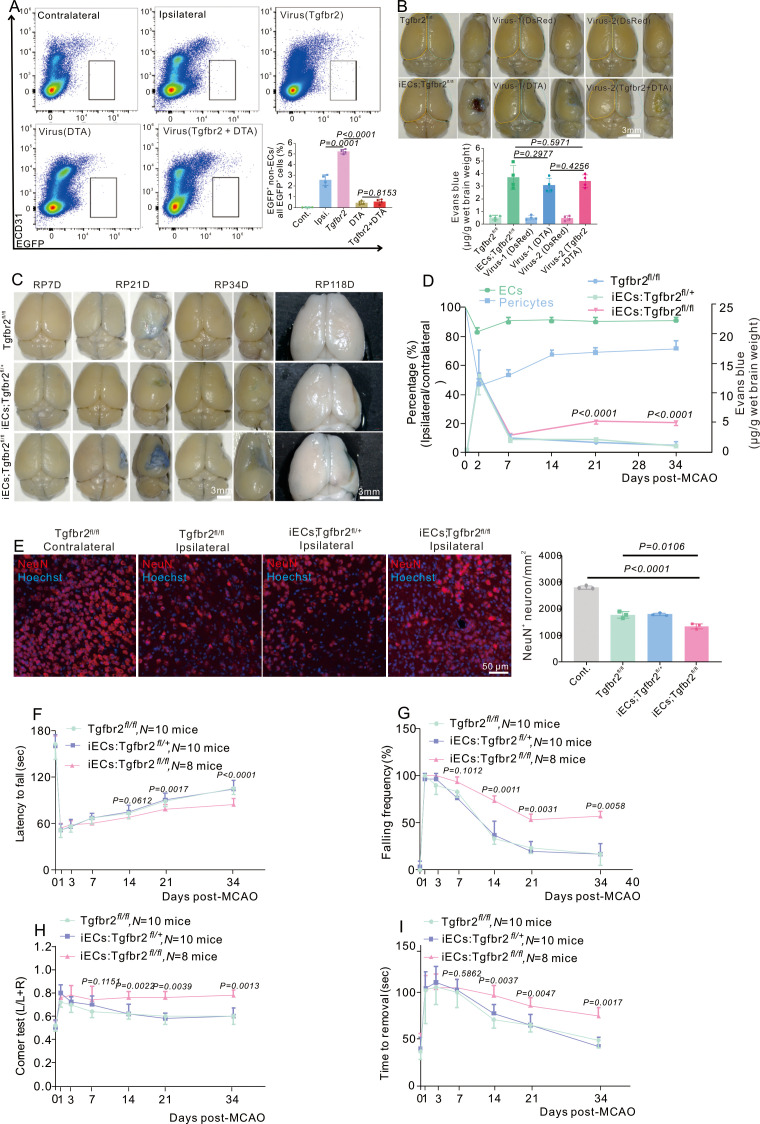


The originally published Figure 9 is shown for reference:

**Figure 2:**
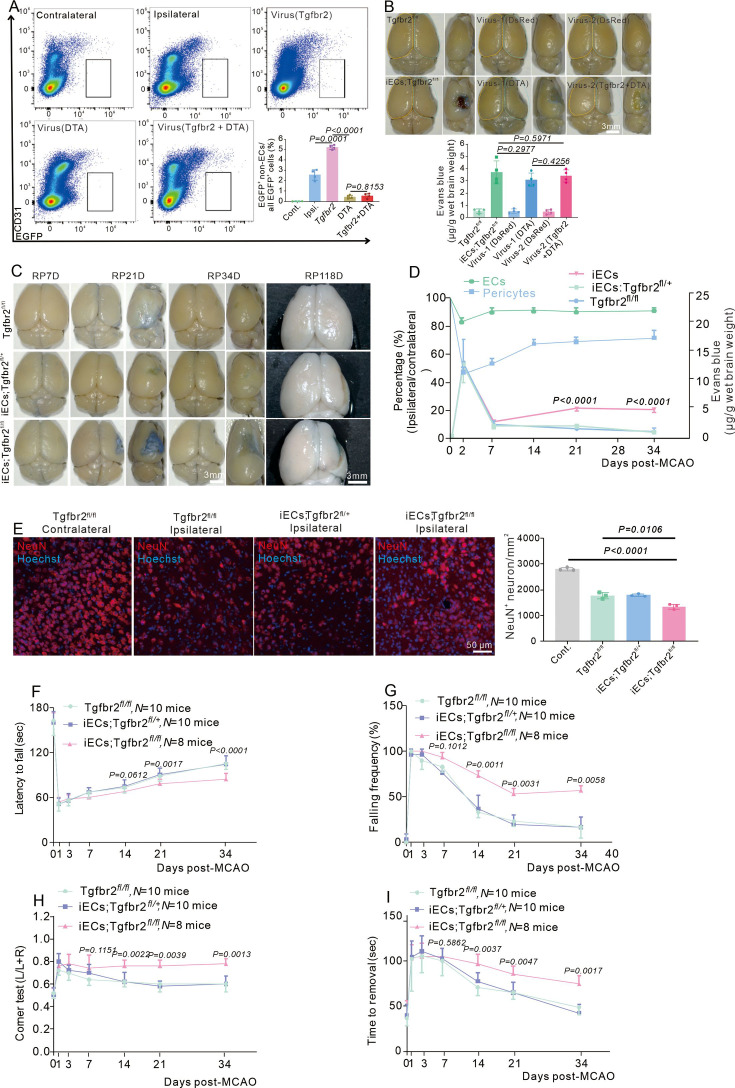


The article has been corrected accordingly.

